# Rhesus Isoimmunization: Late-onset Hemolytic Disease of the Newborn Without Jaundice

**DOI:** 10.7759/cureus.6559

**Published:** 2020-01-04

**Authors:** Maryam Haider, Snober Memon, Fizza Tariq, Sara Fatima, Ammara Hameed

**Affiliations:** 1 Paediatrics, Jinnah Medical and Dental College and Jinnah Medical College Hospital, Karachi, PAK; 2 Paediatrics, Dr. Ruth KM Pfau Civil Hospital, Karachi, PAK; 3 Paediatrics, Civil Hospital, Dow University of Health Sciences, Karachi, PAK; 4 Paediatrics Emergency Medicine, Aga Khan University Hospital, Karachi, PAK; 5 Paediatrics, Bahria University Medical and Dental College, Karachi, PAK

**Keywords:** anemia, blood transfusion, hdn, jaundice, newborn, phototherapy, rh isoimmunization, direct coombs test

## Abstract

Rhesus (Rh) isoimmunization commonly presents with anemia and jaundice of varying intensity in the early postnatal period and is usually treated with phototherapy and exchange transfusion. Rarely, babies with mild or no symptoms at birth may present later with severe hemolytic anemia. This report describes a newborn infant with no postnatal jaundice who presented during the second week of life with severe anemia. These findings indicate the importance of regular follow-up and close monitoring of Rh-isoimmunized infants during the first two months of life for delayed onset anemia.

## Introduction

Hemolytic disease of the newborn (HDN) secondary to Rhesus (Rh) isoimmunization is caused by the transplacental channeling of maternal antibodies active against paternal Rh antigens of the infant and leading to hemolysis. This hemolysis can cause anemia during the intrauterine period and lead to jaundice and anemia of variable intensity after birth. Some neonates with mild or no initial symptoms during the immediate newborn period may subsequently present with late-onset anemia [1]. This report describes a 14-day-old infant born to an Rh-negative mother who presented with late-onset severe anemia in the absence of jaundice.

## Case presentation

A 14-day-old male newborn, weighing 3 kg, born of a nonconsanguineous marriage to a 25-year-old mother (gravida four, para three) by normal vaginal delivery, presented with severe anemia. There were no significant events during the antenatal period. The child was born in good condition, and there were no signs of jaundice, cyanosis, respiratory distress, haematoma, or fits during the immediate postnatal period. On examination, the child had severe anemia, tachypnea, tachycardia, and hepatosplenomegaly. On admission, his hemoglobin was 5.8 g/dL (reference range, 14-24 g/dL); his reticulocyte count (corrected) was 12% (reference range, 1%-2%), and his mean corpuscular volume was 105.3 fL (reference range, 96-108 fL). A peripheral smear showed polychromasia, dimorphic red blood cells (RBCs), target cells, acanthocytes, and spherocytes. His total serum bilirubin concentration was 0.89 mg/dL (reference range, 0.3-1.0 mg/dL), and his direct bilirubin concentration was 0.15 mg/dL (reference range, 0.1-0.3 mg/dL). A full septic work-up was negative for an underlying infectious pathology. The child was strongly positive on a direct Coombs test. Blood typing showed the baby was O-positive, the mother was O-negative, and the father was O-positive. The mother was positive on an indirect Coombs test. The anti-D antibody titers of the mother were also positive, which resulted in a diagnosis of HDN secondary to Rh isoimmunization with anti-D antibodies.

The mother had conceived three times before this pregnancy; all were uneventful normal vaginal deliveries. She had declined anti-D prophylaxis during all her previous pregnancies. All her previous children were born healthy with blood group O-positive and no postnatal complications. The infant diagnosed with HDN was transfused with packed RBCs, but his post-transfusion hemoglobin concentration dropped again to 5.5 g/dL. On day 17, packed RBCs were again transfused, after which his hemoglobin increased slightly to 6.3 g/dL. Two more units of packed RBCs were transfused on days 19 and 22, resulting in a dramatic increase in hemoglobin levels. The infant was finally discharged on day 24 with hemoglobin of 13.6 g/dL and a negative result on a direct Coombs test. The parents were counseled on the nature of their child’s illness, the implications of refusing anti-D prophylaxis, and a high risk of complications during subsequent pregnancies. Contraception was advised. Follow-up visits after one week and monthly for two months showed that the child was thriving well and his hemoglobin concentrations were within the reference range.

## Discussion

Rh isoimmunization is a preventable disease in which an Rh-negative mother develops antibodies against paternal Rh antigen on fetal RBCs. These antibodies are generated by exposure of the fetal blood into the maternal circulation, resulting from fetomaternal hemorrhage secondary to a previous pregnancy, abortion, trauma, or invasive procedure. This phenomenon, called sensitization or iso/alloimmunization, is especially prevalent during an Rh-negative woman’s first pregnancy but is reduced during subsequent pregnancies. Sensitization usually does not affect the firstborn child but can cause hemolytic disease in subsequent newborns. Isoimmunization of Rh-negative pregnant women can be prevented by administering a prophylactic dose of anti-D immunoglobulin, which neutralizes the effect of these isoantibodies. A failure or delay in prophylaxis can destroy fetal RBCs, leading to HDN and is usually treated with phototherapy and exchange transfusion. Although Rh isoimmunization was the main cause of neonatal and perinatal morbidity and mortality for many years, this rate was markedly reduced following the introduction of anti-D immunoglobulin prophylaxis [2,3].

Rh isoimmunization is responsible for neonatal anemia, which varies in severity from fetal hydrops in utero to mild-to-severe anemia during the immediate postnatal period [4]. These isoantibodies may continue to destroy fetal RBCs for six to ten weeks postnatally [5]. Continued destruction may be due to a lack of exchange transfusion during the immediate postnatal period or a progressive improvement in reticuloendothelial function during the second week of life [6,7]. Because of the parallel maturation of the liver, this increased rate of hemolysis may not always be associated with an increase in serum bilirubin concentration [8].

Although the mother was Rh-negative and her previous children were Rh-positive, none of her newborns developed HDN, despite her never receiving anti-D prophylaxis. The lack of HDN during her previous pregnancies may have been due to an intact placental barrier, which may have prevented the mixing of fetal and maternal RBCs, thus avoiding sensitization. This baby, however, developed anemia during the second week of life in the absence of jaundice, an uncommon yet recognized presentation of HDN. Although the baby recovered well after repeated transfusions of packed RBCs, his hospital stay and need for repeated blood transfusions could have been prevented by upfront exchange transfusion at the time of admission.

## Conclusions

The case described here demonstrates that all infants with HDN due to Rh isoimmunization should be considered at risk for late anemia, even in the absence of jaundice or anemia during the immediate postnatal period. Periodic examinations after discharge, including repeat measurements of hemoglobin concentrations, are necessary. Parents should be counseled about the importance of timely medical attention to symptoms of anemia and the necessity of prophylactic anti-D immunoglobulin.
